# Psychological Predictors of Visual and Auditory P300 Brain-Computer Interface Performance

**DOI:** 10.3389/fnins.2018.00307

**Published:** 2018-05-14

**Authors:** Eva M. Hammer, Sebastian Halder, Sonja C. Kleih, Andrea Kübler

**Affiliations:** Institute of Psychology, University of Würzburg, Würzburg, Germany

**Keywords:** predictors, visual P300-BCI, auditory P300-BCI, NVLT, emotional stability

## Abstract

Brain-Computer Interfaces (BCIs) provide communication channels independent from muscular control. In the current study we used two versions of the P300-BCI: one based on visual the other on auditory stimulation. Up to now, data on the impact of psychological variables on P300-BCI control are scarce. Hence, our goal was to identify new predictors with a comprehensive psychological test-battery. A total of *N* = 40 healthy BCI novices took part in a visual and an auditory BCI session. Psychological variables were measured with an electronic test-battery including clinical, personality, and performance tests. The personality factor “emotional stability” was negatively correlated (Spearman's rho = −0.416; *p* < 0.01) and an output variable of the non-verbal learning test (NVLT), which can be interpreted as ability to learn, correlated positively (Spearman's rho = 0.412; *p* < 0.01) with visual P300-BCI performance. In a linear regression analysis both independent variables explained 24% of the variance. “Emotional stability” was also negatively related to auditory P300-BCI performance (Spearman's rho = −0.377; *p* < 0.05), but failed significance in the regression analysis. Psychological parameters seem to play a moderate role in visual P300-BCI performance. “Emotional stability” was identified as a new predictor, indicating that BCI users who characterize themselves as calm and rational showed worse BCI performance. The positive relation of the ability to learn and BCI performance corroborates the notion that also for P300 based BCIs learning may constitute an important factor. Further studies are needed to consolidate or reject the presented predictors.

## Introduction

Brain-Computer Interfaces (BCI) translate intentions into operational commands for technical devices or communication systems without requiring any motor action. Mostly, for non-invasive BCIs, components of the electroencephalogram (EEG) are extracted as input signals. Control signals include event related potentials (ERP) such as the P300 (Farwell and Donchin, 1988; Kleih et al., 2011; Sellers et al., 2012) sensorimotor rhythms (SMR) (Pfurtscheller and Neuper, 1997; Pfurtscheller and McFarland, 2012; Yuan and He, 2014), slow cortical potentials (SCP) (e.g., Birbaumer et al., 1999), or steady state visually evoked potentials (SSVEP) (e.g., Müller-Putz et al., 2005; Allison et al., 2012; Ahn et al., 2015). The ERP P300 is characterized by a positive deflection in the EEG around 300 ms after stimulus onset on central to parietal locations (Polich, 2007) and is elicited by rare deviant stimuli during a stream of frequent standard stimuli, often described as oddball paradigm (Fabiani et al., 1987). In this study we realized a P300 based BCI (further called P300-BCI).

Most P300-BCIs are based on vision; the so-called visual P300 speller was first described by Farwell and Donchin (1988). They presented to their participants a 6 × 6 matrix of characters. To select a letter, the rows and columns of the matrix flashed randomly and the participant had to focus on the target character. By focusing attention on a target, the respective letter turns into a rare stimulus in an oddball paradigm, i.e., the P300 is elicited every time when the target (in the row or column) flashes. For a detailed description of the visual speller (see e.g., Sellers et al., 2012).

For patients with neurodegenerative diseases, such as amyotrophic lateral sclerosis (ALS), who lost the ability to communicate during the progression of the disease, a BCI could be the only remaining possibility to interact with their environment. Preservation of communication is a substantial factor for quality of life (Londral et al., 2015). P300-BCIs are considered end-user friendly because only a short training time is needed to effectively operate the BCI application, high information transfer rates were achieved by end-users with disease (Kaufmann et al., 2013), and they can be used independently of researchers being present at the end-users' home (Sellers et al., 2010; Botrel et al., 2015; Holz et al., 2015).

For patients with impaired vision, visual P300-BCI are not feasible. Auditory BCI may constitute a possible communication channel for such end-users. Several auditory BCI applications were presented using different auditory stimuli instead of flashing rows and columns (Furdea et al., 2009; Höhne et al., 2011; Schreuder et al., 2011b, 2012; Käthner et al., 2013; Simon et al., 2014; Baykara et al., 2016). To provide end users with the BCI that will most likely allow them interaction, it may be beneficial to have predictors of successful BCI operation. End-users could then be tested if they were successful in controlling a BCI. Such a procedure is only feasible if robust predictors can be identified. In the future non-responders might be trained on such predictors as a measure to overcome the BCI inefficiency phenomenon (Kübler et al., 2011).

To date, studies on predictors of P300-BCI performance are sparse. In a sample of 40 healthy BCI users and 11 severely motor impaired end users, Halder et al. (2013a,b, 2016) predicted P300-BCI performance of a web browsing task on the basis of a previously applied auditory oddball. In a sample of healthy participants, a relationship between heart rate variability and BCI performance (visual P300-BCI) was found (Kaufmann et al., 2012). Kleih and colleagues manipulated motivation by monetary reward and showed that the P300 amplitude of highly motivated users was significantly higher as that of less motivated participants (Kleih et al., 2010). Baykara and colleagues also investigated the effects of training and motivation using a multi-class auditory P300 speller in a sample of 16 healthy students (Baykara et al., 2016). Motivation significantly influenced P300 performance rate as well as P300 amplitude. In contrast, the variable “motivation for helping patients” was neither correlated to P300-BCI performance nor to the P300 amplitude (Kleih and Kübler, 2013). But unexpectedly, participants with a low ability for perspective taking, i.e., lower empathy, showed significantly higher P300 amplitudes. Kleih and colleagues speculated that good perspective takers might be emotionally too involved; as a consequence they can hardly concentrate on their actual task, possibly due to a higher workload which was shown to reduce P300 amplitude and P300-BCI performance (Käthner et al., 2014). This effect was also found by Ke and colleagues, but they could also identify a positive impact of mental workload on BCI performance: if participants were exposed to an appropriate extent of mental workload during the BCI training phase, they showed better performances during the following practical application phase (Ke et al., 2016). In comparison, the performance of participants who started under ideal laboratory conditions declined when mental workload increased in a later phase of the study. Sprague and colleagues identified working memory and general intelligence as significant P300-BCI predictors, but working memory failed significance when psychological covariates (e.g., fatigue, mood, motivation) were added into the regression model (Sprague et al., 2016). Nevertheless, this result is in line with Morgan and colleagues, who showed that P300 amplitude decreased when working memory load increased (Morgan et al., 2008). Finally, Gurrera and colleagues reported that the P300 amplitude elicited in an auditory oddball paradigm correlated positively with the personality factors conscientiousness, agreeableness, extraversion, and openness of the Big Five Questionnaire, but negative with neuroticism; correlation coefficients were in the range of 0.38 and 0.51, and thus, moderate to high (Gurrera et al., 2001). It has to be noted that the latter two results used a simple oddball paradigm only, not a P300-BCI. Thus, results cannot be readily transferred to P300-BCI control, but they can serve as a basis for selection of potentially predictive variables.

Taken together little is known about which psychological parameters play a predictive role in BCI control and this is even more so for end-users with disease. This implies that we basically do not know if it is possible to transfer the results of healthy controls to patient end-users. Nijboer et al. (2010) investigated the impact of psychological variables (e.g., depression, motivation, mood) of six ALS patients on their BCI performance (P300-BCI or SMR-BCI). In particular, challenge and mastery confidence—subscales of the Questionnaire for Current Motivation (Rheinberg et al., 2001) adapted to BCI (Nijboer et al., 2008)—were positively correlated with BCI performance, whereas incompetence of fear was negatively related. Current mood showed no correlation with BCI performance. According to Geronimo et al. (2015), ALS patients become less susceptive for using a BCI system with increase of impairment. Importantly, the authors reported that their patients changed their opinion toward the utility of the BCI system as a function of their perceived success during BCI use. Furthermore, they assessed predictors of study participation. Surprisingly, education was identified as a negative predictor. The authors speculated that highly educated patients were still able to work or had other activities which occupied their time. Likewise unexpectedly, high bulbar function was positively related to study participation. Geronimo and colleagues argued that patients with difficulties to speak, may also show more behavioral impairment, which is in turn associated with factors such as apathy and rigidity and hence, lower levels of interest. Patients with cognitive impairment were more interested; however this connection might be due to their cognitive abnormalities because those patients tend to give unrealistically positive judgements.

Recently, we investigated the predictive value of psychological factors on performance with a BCI controlled by SMR (Hammer et al., 2014). We could show that visuo-motor coordination ability and subjects' “attentional impulsivity” accounted for almost 20% of the variance of SMR-BCI performance. Moreover, we were able to identify visuo-motor coordination ability as a predictor for SMR-BCI performance with an average prediction error of 12.07% (Hammer et al., 2012, 2014). Thus, in the current study we were aiming at conducting a comprehensive study of which and to what extent those and other psychological factors can also predict P300-BCI performance.

Following our previous works (Hammer et al., 2012, 2014), in the current study we applied a psychological test-battery including performance, personality, and clinical tests (see section Materials and Methods) to healthy young BCI novices, who subsequently participated in a visual and an auditory P300-BCI session. Our goal was to identify psychological predictors of P300-BCI performance on the basis of the respective results obtained in our previous studies. We assumed that attention, concentration abilities, motivation and conscientiousness would moderately predict performance with the P300-BCI, while other personality traits, other performance variables, intelligence, and clinical variables would show a low or no correlation.

## Materials and methods

The study was conducted at the University of Tübingen, Institute of Medical Psychology and Behavioral Neurobiology, and approved by the Ethical Review Board of the Medical Faculty, Universitiy of Tübingen.

### Participants

A total of 40 healthy BCI novices (normal sighted or with to normal corrected vision; normal hearing; 21 male, 19 female, mean age 25.8, SD 8.46 years, range 17–58) took part in the study. Most participants were students (92%). Each participant gave informed consent after having been informed about the purpose of the study. Participants were paid 8 €/h for their participation. The dataset from one participant had to be excluded from analyses because of technical problems during EEG recording, as well as two further auditory datasets. Thus, *n* = 39 datasets were available for predictor analysis of the visual and *n* = 37 for the auditory P300-BCI.

### Psychological tests

#### Performance tests

##### Cognitrone—COG (Schuhfried, 2007)

The *COG* is a general performance test for the assessment of attention and concentration. Participants had to judge the congruence of a geometrical figure to four reference figures. “Mean time correct rejections” is the mean time the subject needed for a correct rejection when the figure did not match with one of the reference figures.

##### Raven's standard progressive matrices—SPM (Raven, 1998)

The test assesses non-verbal intelligence and logical reasoning. Participants were confronted with a matrix in which one detail was missing. Their task was to choose the matching detail from a set of six or eight choices. Participants were confronted with 60 matrices with increasing complexity. The “total of correct answers” was used as main outcome variable.

##### Verbal learning test—VLT (Sturm and Willmes, 1994b)

The *VLT* assesses verbal learning abilities by presenting neologisms. The participants were instructed that 160 words would be presented and that they would have to memorize them because some words would be recurring during the subsequent test. For each item subjects had to decide if it was new or already seen before. Outcome variables were “sum of correct YES answers,” “sum of incorrect YES answers,” and “sum of the differences of correct minus incorrect YES answers” (difference between all correct and incorrect YES answers).

##### Non-verbal learning test—NVLT (Sturm and Willmes, 1994a)

The *NVLT* assesses non-verbal learning processes by presenting graphical material that is difficult to verbalize. This non-verbal test allows for examination of non-verbal learning abilities and for detection of memory disorders in patients with brain damage. Procedure and outcome variables correspond to those of the Verbal Learning Test (VLT).

#### Personality tests

##### Big five plus one personality-inventory—B5PO (Holocher-Ertl et al., 2003)

The B5PO comprises the six dimensions “empathy,” “emotional stability,” “extraversion,” “conscientiousness,” “openness to experience,” and “agreeableness.” These personality traits were measured on the basis of self-reports. Bipolar adjectives (e.g., quiet vs. active) presented the poles of a response scale. Self-reports were provided via a click with the computer mouse anywhere on the response scale between the bipolar items.

##### Fragebogen zu kontrollüberzeugungen—IPC-scales (Krampen, 1981)

This test assesses generalized locus of control and comprises three scales. A high test value on the “internal scale” *(I-Scale)* means, that participants perceive having control over their own life. The “powerful others scale” *(P-Scale)* assesses the amount of perceived externality due to the subjective feeling of powerlessness. A high value on this scale means that the person feels powerless and that he or she believes to depend on powerful others. The “chance scale” *(C-Scale)* assesses to what extent subjects consider their life dependent on destiny, fortune, misfortune and chance.

##### Achievement motivation-test (Hermans et al., 1978)

The test assesses to what extent subjects attribute success and failure to internal (e.g., ability) or external factors (e.g., fortune) and comprises four scales: “pursuit of accomplishment,” “endurance and diligence,” “exam anxiety that inhibit performance,” and “exam anxiety that supports performance.”

##### Questionnaire for current motivation—QCM (Rheinberg et al., 2001)

Participants' current motivation just before the BCI session was assessed with an adapted (to BCI) version of the “Questionnaire for Current Motivation” (Nijboer et al., 2008), which comprises 18 statements, to be rated on a 7-point Likert-type scale and load on four sub-scales (“mastery confidence,” “fear of incompetence,” “interest,” and “challenge”).

##### Attitudes toward work—AHA (Kubinger and Ebenhöh, 1996)

The *AHA* is an objective personality test which assesses “exactitude,” “decisiveness,” “impulsivity/reflexivity,” “aspiration level,” “performance level,” “frustration tolerance,” “target discrepancy,” and “performance motivation.” The *AHA* comprises three subtests: (1) “Comparing surfaces”: Respondents were asked to choose among three possible answers (right/left/no decision) for deciding about which one of two simultaneously presented surfaces is larger. (2) “Coding symbols”: Participants had to assign symbols to abstract shapes according to a pre-set code, and were asked to estimate their performance in the next task. (3) “Differentiating figures”: Participants were asked to indicate which one of various symbols did not belong to the others.

#### Clinical tests

##### Allgemeine depressionsskala—ADS-L (Hautzinger and Bailer, 1993)

The *ADS-L* is the German version of the Center for Epidemiologic Studies Depression Scale (Radloff, 1977). It is a self-report depression scale designed for the general population. The scale required participants to estimate how much they agree with each of 20 statements on a 4-point Likert-type scale with respect to the last week. Scores range from “0” (best possible) to “60” (worst possible).

##### Symptom checklist-90-revised—SCL90-R (Franke, 1995)

The instrument assesses a broad range of psychological problems and symptoms of psychopathology. The following outcome variables were assessed: “Global Severity Index,” “somatization,” “obsessive-compulsive,” “interpersonal sensitivity,” “depression,” “anxiety,” “hostility,” “phobic anxiety,” “paranoid ideation,” and “psychoticism.”

##### Current mood (Averbeck et al., 1997)

To measure the subjects' current mood during the BCI session, we applied the subscale “current mood” of the “Skalen zur Erfassung der Lebensqualität” (SEL, English: scales to assess quality of life). It comprises 10 items on a 5-point Likert-type scale.

All psychological tests were presented electronically, most of them by the “Vienna Test System (VTS)” which is a computerized psychological assessment tool (Schuhfried GmbH). No computer knowledge was needed to use the system. Answers were entered by the computer mouse or a keyboard. For tests presented via the VTS, the “response panel” (a keyboard that includes joysticks and several buttons) was used.

### Experimental design

Psychological testing and BCI session were conducted on two separate days. Each participant started with the psychological tests, which required about 3 h to be completed. The single BCI session lasted about 4 h, including rest periods. During the BCI session, the participants were seated in a comfortable armchair, ~1 m away from a monitor. Auditory stimuli were presented with conventional headphones. First, an auditory oddball was presented (results see Halder et al., 2013b, data not reported in the current study), followed by the visual P300-BCI, and the auditory P300-BCI paradigms.

#### Visual P300-BCI—the visual speller

We used a 5 × 5 matrix comprising the letters of the Latin alphabet, except the letter Z. The participants' task was to spell the English word “BRAINPOWER” three times, which was presented in two separate words, i.e., in two runs (“Brain” and “Power”). Participants performed six runs (altogether 30 letters) in the so-called copy spelling mode (Kübler et al., 2001), in which they had to spell a pre-set word character by character. Participants were instructed to focus on the target letter and to count how often it flashed or to think “now” whenever the target flashed. No feedback was presented during the first two runs, which served to calibrate the classifier. Then, feedback was provided after each selected character (presented below the target letters).

To select a letter, all subjects were presented with 15 sequences comprising 10 flashes each (one for each row and column) of a duration of 80 ms, followed by an inter-stimulus (ISI) of 160 ms. After each selected letter there was a break of 2.4 s in which the signal was classified and the letter was presented to the BCI user. Accuracy was defined as the overall percentage of correctly selected characters.

#### Auditory P300-BCI—the auditory speller

Following Furdea and colleagues, a so called visual support matrix was provided to the participants to facilitate localizing the target letter; each row and column were represented by a number. The numbers were placed at the top of the columns and at the left side of the rows (Furdea et al., 2009).

In contrast to the visual P300-BCI, rows and columns did not flash, instead the numbers coding the letters were presented acoustically by a male voice. Each character's position was represented by two spoken numbers, the first corresponding to the row and the second to the column. To select a target, participants were instructed to focus on the respective numbers by counting how often the number was presented. First, the numbers of the rows (1–5) and then the numbers of the columns (6–10) were presented. As in the visual paradigm, participants completed six runs, spelling the words “Brain” and “Power” three times, and again no feedback was provided in calibration runs one and two. The stimulus presentation time was 450 ms and ISI was 550 ms. Fifteen sequences were used with intervals of 2.4 s between each letter selection. Again, BCI performance was defined as the percentage of correctly selected letters.

### Data acquisition

The EEG was recorded with Ag/AgCl electrodes in a 128-channel cap (Easycap GmbH), 67 channels (of these, four for vertical and horizontal electrooculography [vEOG/hEOG]) were used during the P300-BCI session. The localization of electrodes was based on an extended 10–20 system of the American Electroencephalographic Society (Sharbrough et al., 1991). Electrode impedances were kept below 5 kΩ. The EEG was recorded with a BrainAmp DC Amplifier (Brainproducts GmbH).

### Preprocessing

Online the EEG data was notch-filtered at 50 Hz and sampled at 500 Hz. The resolution was set to 0.1 μV/bit. This led to an internal sampling rate of 5 kHz which was low-passed at 1 kHz to prevent aliasing. Decimation to 500 Hz was performed within the software of the manufacturer. No additional filtering was applied to the online recording. Before classification, the data was smoothed with a moving average filter with a width of 25 samples and then decimated by a factor of 25.

### Classification

We used stepwise linear discriminant analysis (SWLDA) for online and offline classification (target vs. non-target classification), which is an established classification method for visual and auditory P300-BCI data (e.g., Krusienski et al., 2006).

### Ceiling effect

As described by Halder et al. (2013b), a ceiling effect was observed in the online visual P300-BCI performance (100% accuracy for 28 (70%) participants). Therefore, the data was reclassified offline using all six runs of all participants in a leave-one-run-out cross validation loop and the number of sequences needed to achieve the criterion level for meaningful spelling (70%) (Kübler et al., 2001), was determine, which was three sequences.

### Definition of amplitude and latency

Amplitude of the P300 ERP component was defined as the maximum amplitude between 200 and 800 ms after stimulus presentation. Latency was defined as the time in ms at which the maximum amplitude occurred in relation to stimulus presentation. We chose Cz for the analysis of the visual P300-BCI ERPs and Pz for the analysis of the auditory P300-BCI ERPs because of the more frontal orientation of the visual data (see Halder et al., 2013a, Figure 7).

### Statistical analyses

Normal distributions of data were checked with Kolomogorov-Smirnov tests and with visual inspection of the QQ-Plots. To identify psychological predictors, either Pearson (when variables were normally distributed) or Spearman correlation coefficients (if variables were not normally distributed) were calculated between psychological parameters and P300-BCI performance (correct response rate = CRR [%], i.e., the number of correct letters divided by the total numbers of letters ^*^ 100). For all analyses the respective probability of type I error was maintained at the level of 0.05. For the psychological tests we calculated percentile ranks (PR), standardized for age-independent representative norm samples. If no correspondent norms were available, we used cumulative values. The mean percentile ranks of the measured psychological variables were mainly in a range between PR 16 and PR 84, which indicates average values.

We log transformed the visual P300-BCI CRR because those data were not normally distributed. However, since the auditory P300-BCI performance rates contained zero values, the log transformation was not applicable. Therefore, we transformed the CRR according to van Albada and Robinson to improve the statistical properties (van Albada and Robinson, 2007). To identify psychological variables that could serve as independent variables in the regression, a variable selection procedure was performed: We matched each test to one of the three test groups that were performance, personality or clinical tests (categorization see section Psychological Tests). Then correlations were calculated between CRR and all independent variables from the respective group. Thereby predictors were selected according to the following rule: Variable X is selected if (I) it correlates significantly with the CRR and if (II.) it is not inter-correlated with another variable of the same subgroup that is also correlated with the CRR. If there is an inter-correlation, the variable with the higher correlation coefficient with the CRR was selected. To solve the problem of multiple comparisons, we corrected according to Bonferroni in each block of tests.

To identify significant psychological predictors, we applied linear regression analyses. To determine how much variance in P300-BCI performance could be explained by psychological predictors, we built two regression models, one for the CRR based on visual P300-BCI and one for the CRR based on auditory P300-BCI as dependent variables.

## Results

### P300-BCI online performance

Average visual P300-BCI accuracy was 94.49% (*Md* = 100.0%; *SD* 14.9; 35–100), only two subjects showed poor performance (both 35%). Twenty-eight participants reached 100% CRR. As expected, mean auditory P300BCI performance rate was lower (*M* = 64.32%; *Md* = 85%; *SD* 37.42; 0–100). Seventeen participants (42.5%) remained below the criterion level of 70%.

### Predictor analyses for the visual P300-BCI

After reclassification with three sequences due to the ceiling effect (see section Ceiling Effect) mean CRR of the entire sample was 73.64% (*SD* 22.52; 3–99). All further predictor analyses were conducted with the reanalysed data.

Across all three test groups of tests (performance, personality and clinical), only two outcome variables were significantly correlated with the normalized CRR for the visual P300-BCI: The variable “emotional stability” of the personality test B5PO was negatively correlated (Spearman's rho = −0.416; *p* < 0.01), but failed significance after Bonferroni correction (adjusted alpha level *p* = 0.002).

The outcome variable “sum of the differences of correct minus incorrect YES answers” of the NVLT, which can be interpreted as an ability to learn was positively correlated with the CRR (Spearman's rho = 0.412; *p* < 0.01, adjusted alpha level *p* = 0.006), but also failed significance after Bonferroni correction. “Emotional stability” and the NVLT outcome variable were not inter-correlated (Spearman's rho = −0.021; *p* = 0.899).

To estimate the predictive value of “emotional stability” and the NVLT outcome variable we calculated a linear regression analysis with visual CRR as dependent variable.

The regression of these two variables on visual P300-BCI performance explained about 24% of the variance [*R*^2^ = 0.242; *F*_(2, 36)_ = 5.74; *p* < 0.05]. On its own, “emotional stability” accounted only for 5% (*R*^2^ = 0.049; *F*_(1, 37)_ = 1.91; *p* = 0.175] of the variance and failed significance. The NVLT variable was identified as a significant predictor which accounted for about 19% [*R*^2^ = 0.189] of the variance. Key parameters of the regression model are listed in Table 1.

Table 1Model summary and significance tests of the regression model corresponding to the variables “emotional stability” and “sum of the differences of correct minus incorrect YES answers.”

**Table:** 

**Model**	***R*^2^**	**ANOVA**					
			**Sum of squares**	***df***	**Mean square**	***F***	**Sig**.
emot. stability	0.049	Regression	0.79	1	0.79	1.91	0.175^a^
		Residual	15.32	37	0.41		
		Total	16.11	38			
emot. stability + SUMD	0.242	Regression	3.89	2	1.95	5.74	0.007^b^
		Residual	12.22	36	0.34		
		Total	16.11	38

**Table:** 

**Coefficients**					
	**Unstandardized coefficients**		**Standardized coefficients**		
**Model**	***B***	**Std. error**	**Beta**	***t***	**Sig**.
1 (constant)	1.984	0.128		15.456	0.000
emotional stability	−0.002	0.002	−0.221	−1.381	0.176
2 (constant)	1.673	0.155		10.778	0.000
emotional stability	−0.002	0.002	−0.230	−1.583	0.122
SUMD	0.005	0.001	0.439	3.024	0.005

a *Independent variable: “emotional stability” (BIG5PO)*.

b *Independent variables: “emotional stability” (BIG5PO), “sum of the differences of correct minus incorrect YES answers” (NVLT). Dependent variable: visual P300-BCI performance*.

**Figure 1:**
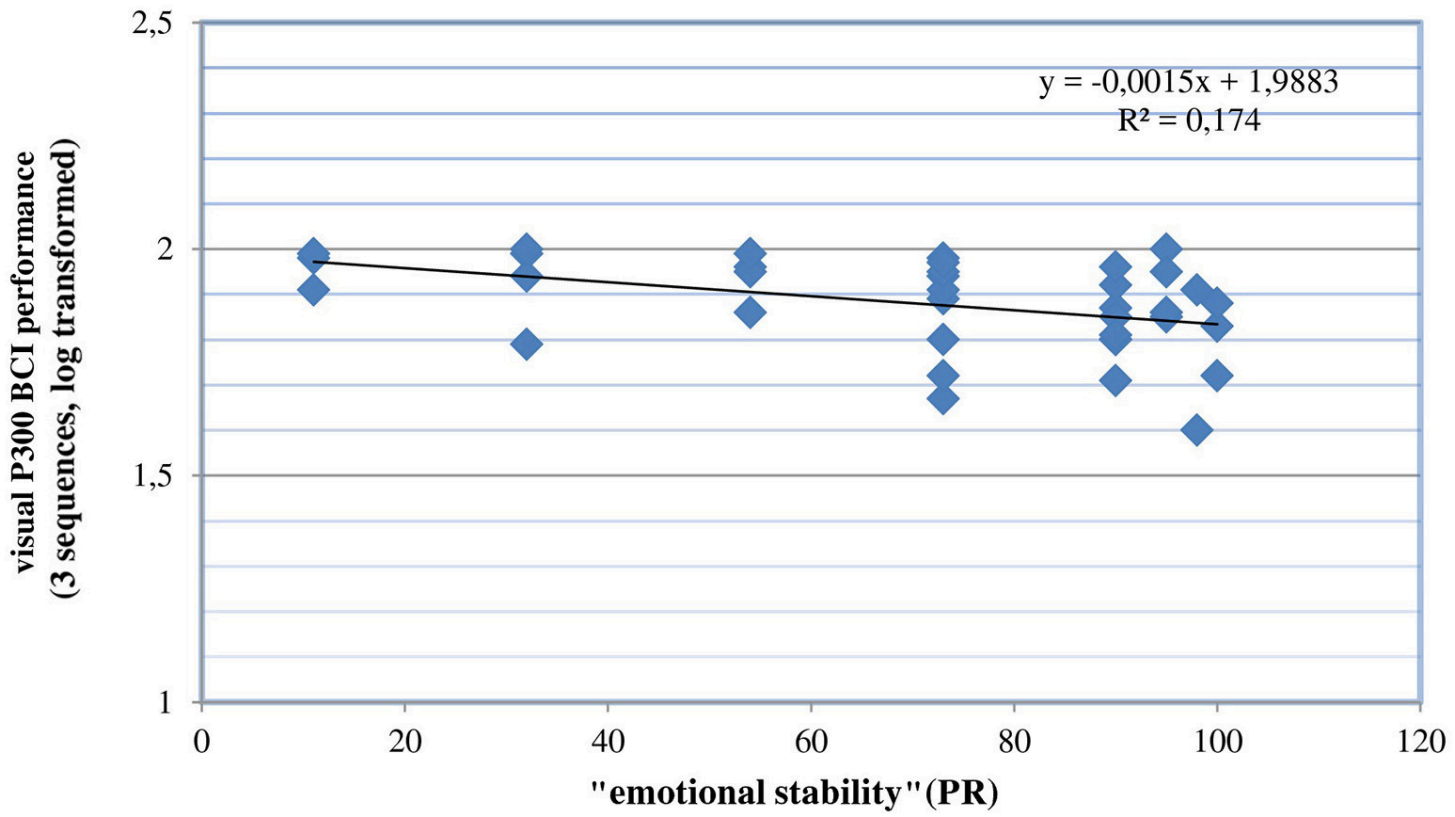
Correlation between emotional stability and the visual P300-BCI offline performance after two outlier values were excluded (PR is the abbriviation for percentile rank).

**Figure 2:**
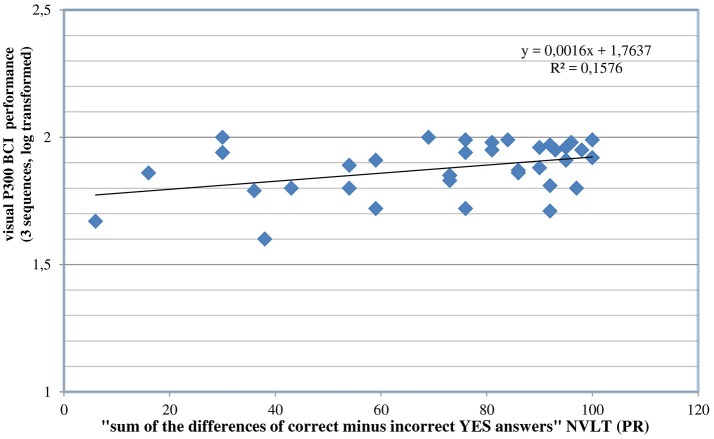
Correlation between “sum of the differences of correct minus incorrect YES answers” (NVLT; = “the ability to learn”) and the visual P300-BCI performance after excluding two outlier values.

By visual inspection of the data, two outliers were identified, namely two participants whose CRR were poor (both 35%). To check the influence of these outlier values on the regression model, we excluded both and conducted a new regression analysis. The entire regression model explained about 36% of the variance [*R*^2^ = 0.361; *F*_(2, 34)_ = 9.62; *p* < 0.001], “emotional stability” explained about 17% of the variance [*R*^2^ = 0.169; *F*_(1, 35)_ = 7.13; *p* < 0.05], both variables were identified as significant predictors (see Figures 1, 2).

### Predictor analysis for auditory P300-BCI

Again, “emotional stability” was significantly correlated with the normalized auditory P300-BCI CRR (Spearman's rho = −0.377; *p* < 0.05, adjusted alpha level *p* = 0.002), but the correlation failed significance after Bonferroni correction. No other significant correlations were found. We calculated a linear regression analysis with “emotional stability” as independent variable and the auditory P300-BCI CRR as dependent variable. The independent variable explained 8% of the variance (*R*^2^ = 0.084) but failed significance. Key parameters of the regression model are listed in Table 2.

Table 2Model summary and significance tests of the regression model corresponding to the variable “emotional stability.”

**Table:** 

**Model**	***R*^2^**	**ANOVA**					
			**Sum of squares**	***df***	**Mean square**	***F***	**Sig**.
Emotional stability	0.084	Regression	2.12	1	2.12	33.19	0.083^a^
		Residual	23.24	35	0.664		
		Total	25.36	36	Total

**Table:** 

**Coefficients**					
	**Unstandardized coefficients**		**Standardized coefficients**		
**Model**	***B***	**Std. error**	**Beta**	***t***	**Sig**.
1 (constant)	0.437	0.408		1.069	0.292
emotional stability	−0.009	0.005	−0.289	−1.787	0.083

a *Independent variable: “emotional stability” (BIG5PO); Dependent variable: auditory P300-BCI performance*.

### Relation between psychological and physiological variables

**Figure 3:**
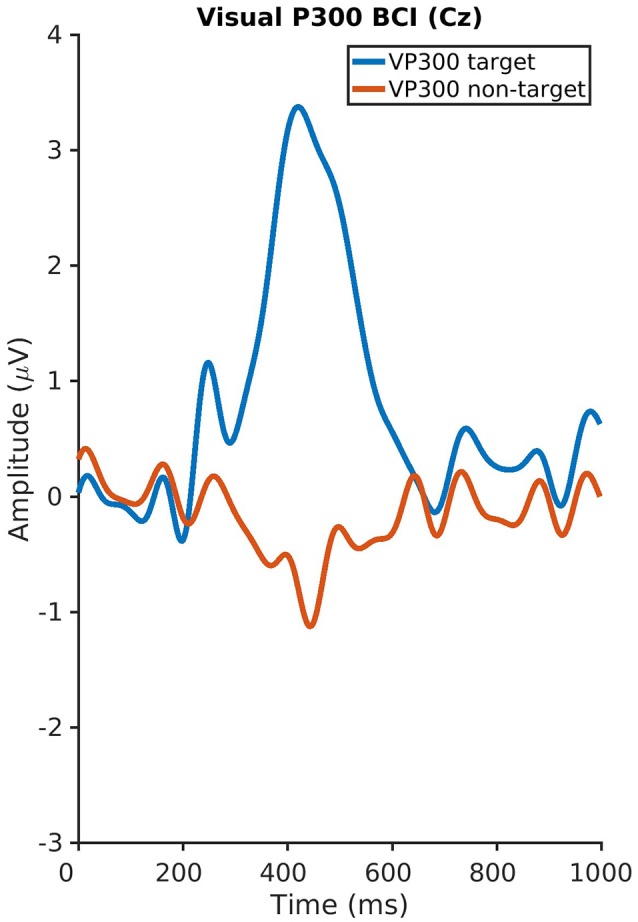
Plot of the visual P300 at Cz averaged across all subjects.

**Figure 4:**
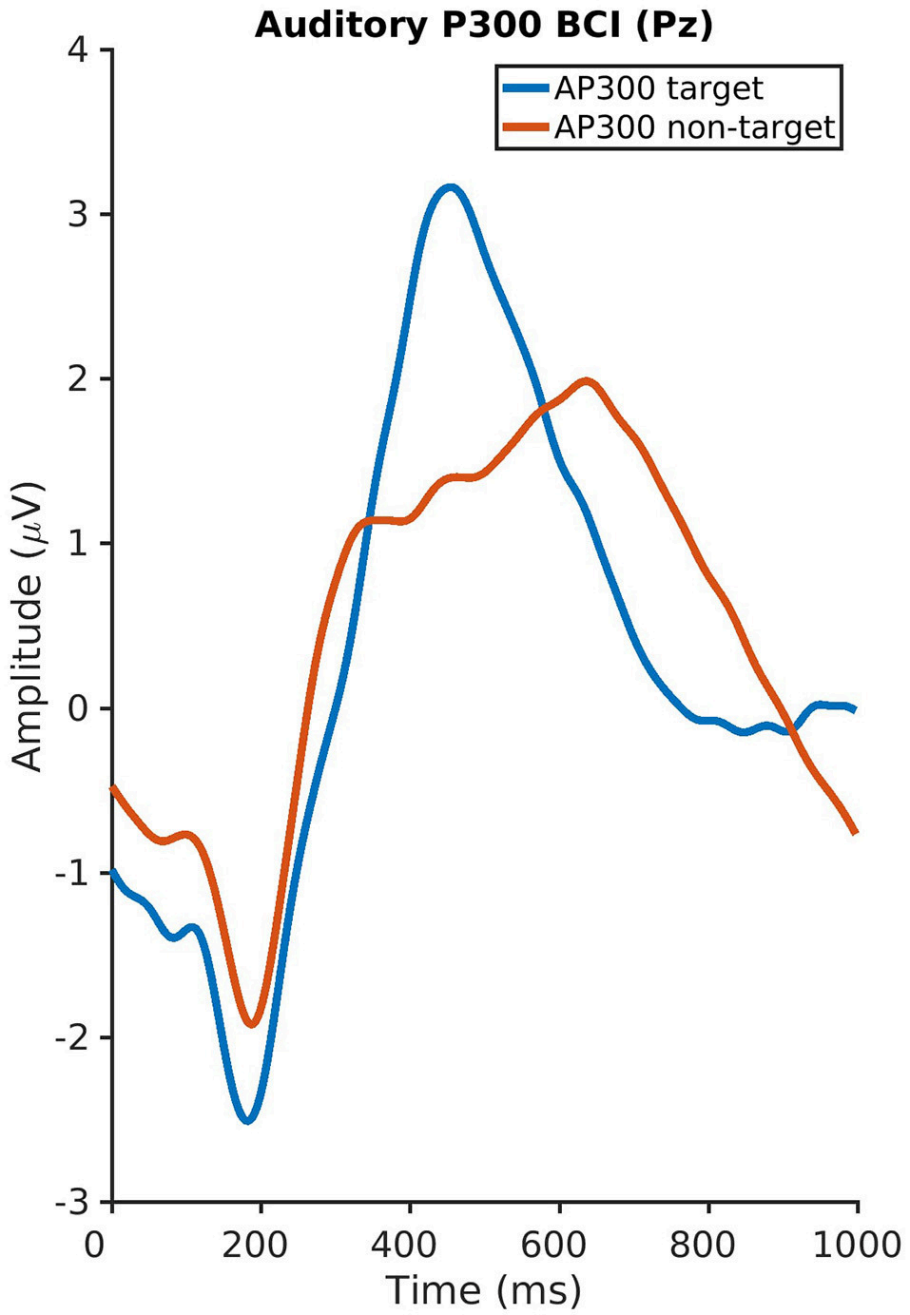
Plot of the auditory P300 at Pz averaged across all subjects.

The auditory P300 ERP component had a mean amplitude of 4.8 μV (*SD* 3.2, range 0.7–11.1) and a latency of 471 ms (*SD* 128, range 210–800) and the visual P300 a mean amplitude of 4.1 μV (*SD* 2.6, range 1.4–12.2) and latency of 432 ms (*SD* 109, range 240–800). No significant correlations were found between the psychological variables and the P300 amplitudes and latencies neither for the visual nor for the auditory P300-BCI. Plots of the visual and auditory P300 at Cz and Pz averaged across all subjects are depicted in Figures 3, 4.

## Discussion

The purpose of the current study was to investigate psychological variables that may serve as performance predictors in a P300-BCI with visual and auditory stimulation. On the basis of previous results, we assumed that attention, concentration abilities, motivation, and conscientiousness would moderately predict performance with the P300-BCI, while other personality traits, other performance variables, intelligence, and clinical variables would show low or no correlation.

In the visual P300-BCI, two variables were related to BCI performance: “Emotional stability,” a variable of the B5PO, was negatively correlated and the variable “sum of the differences of correct minus incorrect YES answers” of the NVLT was positively correlated with visual P300-BCI performance. After exclusion of two outlier values, both variables were identified as significant predictors, explaining about 36% of the variance; on its own “emotional stability” explained about 17% of the variance. “Emotional stability” was also negatively correlated with auditory P300-BCI performance and explained 8% of the variance. This result replicates that of the visual P300-BCI albeit the explained variance was much lower.

The personality factor “emotional stability” is often referred to as the low pole of the personality trait neuroticism. People with high a score on the factor “emotional stability” characterize themselves as calm, withdrawn and rational compared to frantic, impulsive and emotional. Highly neurotic people report higher anxiety values and feel more stressed and unconfident. Unexpectedly, participants with a high score of “emotional stability” showed worse BCI performance, in our study. To date, there have been no BCI studies that found a correlation between “emotional stability” and BCI performance, but there are some studies that attributed a negative impact of high neuroticism on attention and information processing. Consistently, high neuroticism was associated with cognitive inflexibility and difficulty in ignoring irrelevant stimuli (Maclean and Arnell, 2010) as well as difficulties in disengaging from salient stimuli, meaning high neurotic people tend to overinvest attentional resources in the processing of salient yet irrelevant stimuli (Dhinakaran et al., 2014). Greater neuroticism is associated with a longer attentional blink (a short attention deficit occurring in rapid serial visual presentation tasks), which is connected to less efficient cognitive control (Maclean and Arnell, 2010). Fjell et al. (2005) aimed at describing differences in information processing by comparing a group of highly stable and a group of highly neurotic participants on the basis of event-related potentials. They did not find differences in an auditory P300 and a visual P300 oddball task. The authors concluded that there is no significant disparity in fast neurocognitive processing between highly emotionally stable and highly neurotic people. To summarize, our findings contradict results reported in the literature. One reason may be that, in contrast to those studies, the group of highly neurotic subjects was rather small in the current sample—only three participants showed highly neurotic test values with a PR smaller than 16. Eysenck postulated in his arousal theory (Eysenck, 1967) that people with higher neuroticism scores have lower excitation thresholds in subcortical structures which would be associated with a higher level of arousal of the limbic system. Hence, one might speculate that BCI users with higher neuroticism values were more responsive to the given task because of their lower arousal thresholds. Potentially, higher neuroticism turned into a small advantage for operating a P300-BCI. The fact that “emotional stability” was identified as predictor in both P300-BCI paradigms cautiously supports its validity, which of course requires further investigation. To test the stability of the newly found predictor in a further study, one could first identify participants with extreme values on the two poles of the dimension neuroticism and then compare P300-BCI performance between the two groups in a second step.

The predictor “sum of the differences of correct minus incorrect YES answers” can be interpreted as an ability to learn. The *NVLT* assesses non-verbal learning processes—which may be considered relevant to master the P300-BCI tasks—by presenting graphical material that is difficult to verbalize. A high score of the outcome variable “sum of the differences of correct minus incorrect YES answers” means that a participant is able to differentiate between new and already presented items. Therefore, it is also an indicator of memory capacity and the ability to concentrate on the presented material, and both memory and concentration are mandatory for learning. Sachs et al. (2004) investigated anomalies in ERPs in patients with the diagnosis social phobia. They identified reduced P300 amplitude and increased P300 latency. The latter was correlated with deficient learning abilities measured by the NVLT supporting the potential relevance of this variable for P300-BCI performance. Our findings are in line with the results of Sprague and colleagues, who identified working memory as a significant predictor of P300-BCI performance (Sprague et al., 2016). They proposed that working memory could be improved throughout a special training program with the aim to increase BCI performance. The authors discussed different experimental designs how one could test the effectivity of a training program. They also stated that long-term repeated BCI use itself could lead to an improvement in working memory. Therefore, they recommended comparing three groups: a working memory training group, a BCI group and a control group. Such a study design could also be used to investigate the causal effect of non-verbal learning abilities on P300-BCI performance. It is important to note, that we—at the time the study was conducted—did not expect the “ability to learn” to be relevant for P300-BCI performance. Specifically, healthy subjects are often at 100% CRR in the first session (Guger et al., 2009). However, recently it was shown that also P300-BCI performance can increase with learning, specifically under more difficult conditions. Baykara and colleagues and Herweg and Kübler confronted healthy subjects with non-visual BCIs and demonstrated a significant learning in the auditory and the tactile modality (Baykara et al., 2016; Herweg et al., 2016). Halder and colleagues confirmed these results in end-users with neurodegenerative disease (Halder et al., 2016). These results indicate that the “ability to learn” may be more relevant for P300-BCI than previously assumed.

In contrast to Sprague et al. (2016) intelligence was not identified as a significant predictor in the current study. This may be attributed to different definitions of intelligence. Sprague and colleagues used the *Picture Vocabulary Test*, which measures general intelligence, specifically crystallized intelligence, whereas the *Raven's Standard Progressive Matrices (SPM)*, which was used in the current study, assesses fluid intelligence.

Unlike Kleih and colleagues and Baykara and colleagues motivation was not identified as a significant predictor in the current study (Kleih et al., 2010; Baykara et al., 2016). Differences in study designs may account for this discrepancy. Motivation was systematically manipulated by Kleih and colleagues and Bayakara and colleagues used animal sounds in their auditory P300-BCI and subjects had to accomplish five BCI sessions.

Against our assumption, we could not identify a significant correlation between attention or concentration assessed via the Cognitrone test and the P300-BCI performance. The Cognitrone provides information about selective attention abilities, meaning focusing attention on relevant stimuli while ignoring irrelevant aspects of the task. These skills are substantial for controlling effectively a P300-BCI. A recent study showed that changes in stimulus presentation, such that they better catch the users' attention (honeycomb-shaped figure with red dots), lead to improved BCI performance (Jin et al., 2017). Lakey et al. (2011) tried to manipulate attention through a short mindfulness meditation induction. Participants of the treatment group showed higher P300-BCI performance rate as well as larger P300 amplitudes compared to BCI users of the control group. Thus, attentional processes play an important role for P300-BCI control, but it was not possible to operationalize this effect with the Cognitrone in our study. Concerning BCI end users, Geronimo et al. (2016) reported that ALS patients become less effective at utilizing a BCI system with increase in cognitive impairment, particularly in the realm of attention.

In contrast to our hypothesis, the personality factor conscientiousness was not linked to performance. Albeit we found an effect of two psychological variables on P300-BCI performance, none of the psychological variables were related to the amplitude and latency of the P300. This may be explained by the high number of sequences in the online session which reduces the variance of the P300 amplitude. It has to be noted that we also could not find any predictors for the visual P300-BCI performance when using online performance as dependent variable, due to a lack of variance in the data. In further studies, the number of sequences has to be reduced, e.g., such that an online performances of 70% CRR is achieved.

There are several limitations of the current study: As in many other BCI studies, we recruited a sample of young and healthy people, who had high a level of education. These sample characteristics lead to reduced variance in psychological test results, which may be responsible for the lack of significant correlations. Further, it is not possible to generalize these results to BCI end users with disease, because there are several differences between those groups (e.g., age distribution, cerebral gray, and white matter volume). For example, McCane et al. (2015) compared patients with ALS and age-matched controls with respect to their performance in a visual P300-BCI and amplitude, latency, and location of other potentially relevant ERPs. The authors reported that the groups did not differ in P300-BCI performance or information transfer rate. Differences were observed in the location of the target ERPs, the amplitudes of the late positivity, the amplitude of the early negativity (N200) and the latency of the late negativity. These results emphasize the necessity of studies with patients in order to investigate reliability and validity of predictors that were identified for healthy BCI-users.

Due to the large number of psychological test variables, e.g., the subgroup of personality tests comprised 25 outcome variables, the sample size was too small to maintain significance after Bonferroni correction. Thus, to gain higher power a larger sample must be included which, however, increases the effort for data collection. Finally, our number of stimulus repetitions was too high such that we encountered the described ceiling effect and needed to use offline data for the predictor analysis. At the time of data collection the dynamic stopping method (Schreuder et al., 2011a, 2013; Kindermans et al., 2014) was not yet published, which nowadays should be included in such an approach. This holds also true for the by now established face overlay to elicit larger a P300 and additional ERPs (N170 and N400f; Kaufmann et al., 2013; Zhou et al., 2016).

## Conclusions

There are many psychological factors—personality, performance, and clinical—that could potentially influence P300-BCI performance. The current study identified only few that indeed did so and these were “emotional stability” and the “ability to learn.” Emotional stability negatively predicted P300-BCI performance in both modalities, which is counter-intuitive and contradicts other findings. However, as it was found in both paradigms—auditory and visual—it is worth further investigation. The significant predictor for the “ability to learn” is also somewhat surprising as learning has—until recently—not been assumed as particularly relevant for operating a P300-BCI. Yet, recent results with non-visual P300-BCIs and end-users with disease point toward learning as relevant when conditions are more difficult. As we found the “ability to learn” only predictive of visual P300-BCI performance, also this predictor requires further studies for its confirmation or rejection. Taken together, in healthy subjects only few psychological factors seem to play a moderate role for P300-BCI performance. To confirm or reject these predictors, studies with more difficult conditions, such as few sequences, non-visual modalities or distracting additional tasks, are necessary. To establish causality, experimental designs are required that manipulate the predicting variables.

## Ethics statement

This study was carried out in accordance with the recommendations of the Ethical Review Board of the Medical Faculty of the University of Tübingen with written informed consent from all subjects. All subjects gave written informed consent in accordance with the Declaration of Helsinki. The protocol was approved by the Ethical Review Board.

## Author contributions

EH: Study concept, data assessment, data analyses and preparation of the manuscript; SH: Data assessment, data analyses; SK: Data assessment; AK: Study conception, data analyses and preparation of the manuscript supervised.

### Conflict of interest statement

The authors declare that the research was conducted in the absence of any commercial or financial relationships that could be construed as a potential conflict of interest.
